# 3,3′-(2,2′-Bi-1*H*-imidazole-1,1′-di­yl)dipropanamide

**DOI:** 10.1107/S1600536809028955

**Published:** 2009-07-29

**Authors:** Y.-X. Zhi, J. Long, J.-Y. Chen, Y.-T. Ren

**Affiliations:** a Gemmological Institute, China University of Geosciences, Wuhan, Hubei 430074, People’s Republic of China; b China University of Geosciences, Engineering Research Center of Nano-Geomaterials of the Ministry of Education, Wuhan, Hubei 430074, People’s Republic of China

## Abstract

In the title compound, C_12_H_16_N_6_O_2_, the two imidazole rings are coplanar as a center of inversion exists midway along the C—C bond joining the two rings. In the crystal, inter­molecular N—H⋯O, N—H⋯N and C—H⋯O hydrogen bonds link adjacent mol­ecules into a two-dimensional layer structure parallel to (001).

## Related literature

For the coordination chemistry and biological activity of bis-imidazoles, see: Kirchner & Krebs (1987 ▶); Tadokoro *et al.* (1999 ▶).
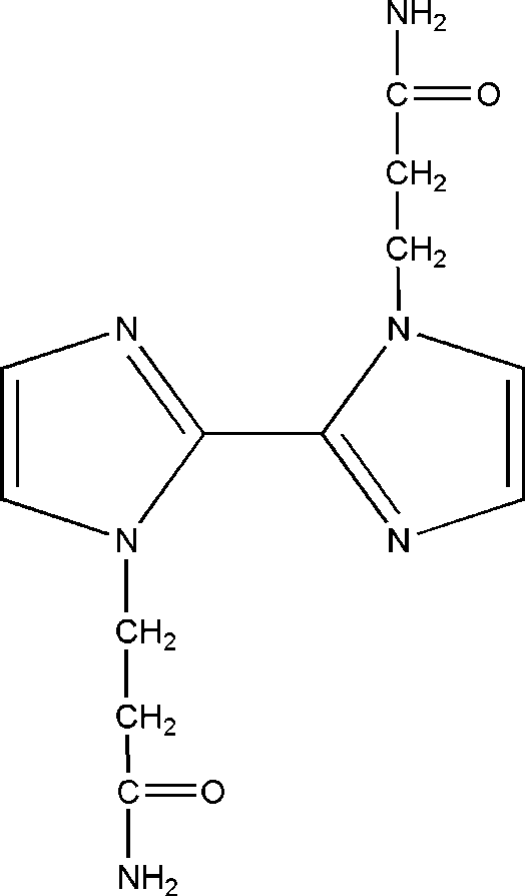


## Experimental

### 

#### Crystal data

C_12_H_16_N_6_O_2_
*M* *_r_* = 276.31
Monoclinic, 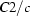

*a* = 18.445 (4) Å
*b* = 4.8622 (10) Å
*c* = 13.446 (3) Å
β = 93.38 (3)°
*V* = 1203.8 (5) Å^3^
*Z* = 4
Mo *K*α radiation
μ = 0.11 mm^−1^
*T* = 295 K
0.58 × 0.46 × 0.20 mm

#### Data collection

Rigaku R-AXIS RAPID diffractometer
Absorption correction: multi-scan (*ABSCOR*; Higashi, 1995 ▶) *T*_min_ = 0.936, *T*_max_ = 0.980
4987 measured reflections
1381 independent reflections
1237 reflections with *I* > 2σ(*I*)
*R*_int_ = 0.017

#### Refinement

*R*[*F* ^2^ > 2σ(*F* ^2^)] = 0.045
*wR*(*F* ^2^) = 0.111
*S* = 1.22
1381 reflections
92 parameters
H-atom parameters constrained
Δρ_max_ = 0.33 e Å^−3^
Δρ_min_ = −0.28 e Å^−3^

### 

Data collection: *RAPID-AUTO* (Rigaku, 1998 ▶); cell refinement: *RAPID-AUTO*; data reduction: *CrystalStructure* (Rigaku/MSC, 2002 ▶); program(s) used to solve structure: *SHELXS97* (Sheldrick, 2008 ▶); program(s) used to refine structure: *SHELXL97* (Sheldrick, 2008 ▶); molecular graphics: *SHELXTL* (Sheldrick, 2008 ▶); software used to prepare material for publication: *SHELXL97*.

## Supplementary Material

Crystal structure: contains datablocks global, I. DOI: 10.1107/S1600536809028955/ng2617sup1.cif

Structure factors: contains datablocks I. DOI: 10.1107/S1600536809028955/ng2617Isup2.hkl

Additional supplementary materials:  crystallographic information; 3D view; checkCIF report

## Figures and Tables

**Table 1:** Hydrogen-bond geometry (Å, °)

*D*—H⋯*A*	*D*—H	H⋯*A*	*D*⋯*A*	*D*—H⋯*A*
N3—H3*A*⋯N2^i^	0.86	2.22	3.055 (1)	164
N3—H3*B*⋯O1^ii^	0.86	2.13	2.967 (2)	165
C5—H5*B*⋯O1^ii^	0.97	2.58	3.293 (3)	130

Symmetry codes: (i) 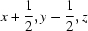; (ii) 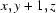.

**Fig. 1.**
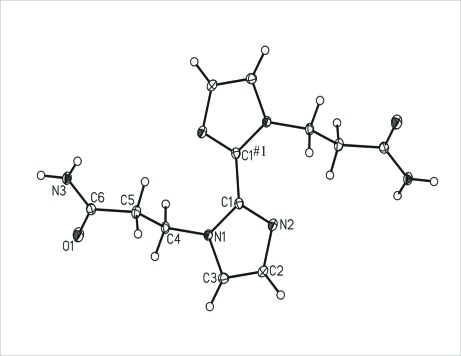
View of the molecular structure of the title compound, Displacement ellipsoids are drawn at the 45% probability level.[Symmetry codes: (i) -x + 1/2, -y + 3/2, -z + 1]

## supplementary crystallographic information

### Comment

As part of our ongoing investigations, the title compound, *L*^3^, C_12_H_16_N_6_O_2_, as a derivative of 2,2'-bimidazole whose compounds were abstacted for their coordination chemistry and biological activity (Kirchner *et al.*, 1987; Todokoro *et al.*, 1999), has been synthesized and structurally characterized. The single imidazole ring exhibits nearly perfect coplanarity with the maximal deviation of 0.001 (1) Å and the two imidazole rings are coplanar. There are intermolecular N—H···N, N—H···O, C—H···O and C—H···N hydrogen bonds, which leads to two-dimensional layers parallel to (001). Eventually, the crystal packing is established by van der Waals forces.

### Experimental

A solution of acrylamide (14.2 g, 0.20 mol) in 50 ml DMF was dropwise added to a stirred suspension of 2,2'-biimidazole (13.4 g, 0.1 mol) and NaOH (0.8 g, 0.02 mol) in 100 ml DMF at 80°C, the colour of the resulting solution varied from colourless through green to orange. After the mixture was refluxed for six hours, the crude product was obtained by removement of DMF solvent under reduced pressure. The product was isolated,washed by 10 ml aether for three times, and then dried *in vacuo* to give the pure compound *L*^3^ in a 74.3% yield. Colourless single crystals of *L*^3^ suitable for single X-ray analysis were recrystallized by slow evaporation of a deionized aqueous solution.^1^H NMR (400 MHz, D_2_O, 25°C, TMS, p.p.m.) δ: 8.402(s, 4H), 7.306(s, 2H), 7.140(s, 2H), 4.374(s, 4H), 2.627(s, 4H). ^13^C NMR (400 MHz, D_2_O, 25°C, TMS, p.p.m.) δ:171.53, 136.57, 128.15, 122.39, 42.96, 35.06. IR (KBr, cm^-1^): 3388*m*, 1674 s, 1409 s, 1267 s, 769 s. Anal. Calcd for *L*^3^ (%): C, 52.17; H, 5.80; N, 30.22. Found: C, 52.12; H, 5.70; N, 29.89.

### Refinement

H atoms bonded to C atoms were palced in geometrically calculated position and were refined using a riding model, with *U*_iso_(H) = 1.2 *U*_eq_(C). H atoms attached to O atoms were found in a difference Fourier synthesis and were refined using a riding model, with the O—H distances fixed as initially found and with *U*_iso_(H) values set at 1.2 *U*eq(O).

### Crystal data

**Table:**

C_12_H_16_N_6_O_2_	*F*(000) = 584
*M**_r_* = 276.31	*D*_x_ = 1.525 Mg m^−^^3^
Monoclinic, *C*2/*c*	Mo *K*α radiation, λ = 0.71073 Å
Hall symbol: -C 2yc	Cell parameters from 1381 reflections
*a* = 18.445 (4) Å	θ = 3.0–27.5°
*b* = 4.8622 (10) Å	µ = 0.11 mm^−^^1^
*c* = 13.446 (3) Å	*T* = 295 K
β = 93.38 (3)°	Platelet, colorless
*V* = 1203.8 (5) Å^3^	0.58 × 0.46 × 0.20 mm
*Z* = 4	

### Data collection

**Table:**

Rigaku R-AXIS RAPID diffractometer	1381 independent reflections
Radiation source: fine-focus sealed tube	1237 reflections with *I* > 2σ(*I*)
graphite	*R*_int_ = 0.017
ω scans	θ_max_ = 27.5°, θ_min_ = 3.0°
Absorption correction: multi-scan (*ABSCOR*; Higashi, 1995)	*h* = −23→23
*T*_min_ = 0.936, *T*_max_ = 0.980	*k* = −6→6
4987 measured reflections	*l* = −15→17

### Refinement

**Table:**

Refinement on *F*^2^	Secondary atom site location: difference Fourier map
Least-squares matrix: full	Hydrogen site location: inferred from neighbouring sites
*R*[*F*^2^ > 2σ(*F*^2^)] = 0.045	H-atom parameters constrained
*wR*(*F*^2^) = 0.111	*w* = 1/[σ^2^(*F*_o_^2^) + (0.0012*P*)^2^ + 5.254*P*] where *P* = (*F*_o_^2^ + 2*F*_c_^2^)/3
*S* = 1.22	(Δ/σ)_max_ < 0.001
1381 reflections	Δρ_max_ = 0.33 e Å^−^^3^
92 parameters	Δρ_min_ = −0.28 e Å^−^^3^
0 restraints	Extinction correction: *SHELXL97* (Sheldrick, 2008), Fc^*^=kFc[1+0.001xFc^2^λ^3^/sin(2θ)]^-1/4^
Primary atom site location: structure-invariant direct methods	Extinction coefficient: 0.0061 (5)

### Special details

**Table:**

Geometry. All e.s.d.'s (except the e.s.d. in the dihedral angle between two l.s. planes) are estimated using the full covariance matrix. The cell e.s.d.'s are taken into account individually in the estimation of e.s.d.'s in distances, angles and torsion angles; correlations between e.s.d.'s in cell parameters are only used when they are defined by crystal symmetry. An approximate (isotropic) treatment of cell e.s.d.'s is used for estimating e.s.d.'s involving l.s. planes.
Refinement. Refinement of *F*^2^ against ALL reflections. The weighted *R*-factor *wR* and goodness of fit *S* are based on *F*^2^, conventional *R*-factors *R* are based on *F*, with *F* set to zero for negative *F*^2^. The threshold expression of *F*^2^ > σ(*F*^2^) is used only for calculating *R*-factors(gt) *etc*. and is not relevant to the choice of reflections for refinement. *R*-factors based on *F*^2^ are statistically about twice as large as those based on *F*, and *R*- factors based on ALL data will be even larger.

### Fractional atomic coordinates and isotropic or equivalent isotropic displacement parameters (Å^2^)

**Table:**

	*x*	*y*	*z*	*U*_iso_*/*U*_eq_	
O1	0.47285 (8)	0.1364 (3)	0.37688 (12)	0.0174 (4)	
N3	0.52500 (9)	0.5591 (4)	0.38322 (12)	0.0125 (4)	
H3A	0.5683	0.4933	0.3846	0.015*	
H3B	0.5184	0.7341	0.3846	0.015*	
C3	0.21467 (11)	0.3911 (4)	0.33180 (15)	0.0120 (4)	
H3C	0.2203	0.2520	0.2854	0.014*	
C4	0.33714 (10)	0.3479 (4)	0.41994 (15)	0.0112 (4)	
H4A	0.3370	0.1679	0.3888	0.013*	
H4B	0.3497	0.3241	0.4905	0.013*	
N1	0.26419 (9)	0.4700 (4)	0.40652 (12)	0.0100 (4)	
C6	0.46787 (11)	0.3892 (4)	0.37902 (14)	0.0112 (4)	
C2	0.15558 (10)	0.5566 (4)	0.33885 (15)	0.0119 (4)	
H2A	0.1136	0.5477	0.2971	0.014*	
C1	0.23297 (10)	0.6812 (4)	0.45616 (14)	0.0097 (4)	
N2	0.16681 (9)	0.7385 (4)	0.41640 (13)	0.0117 (4)	
C5	0.39394 (10)	0.5287 (4)	0.37442 (15)	0.0122 (4)	
H5A	0.3789	0.5678	0.3055	0.015*	
H5B	0.3976	0.7020	0.4101	0.015*	

### Atomic displacement parameters (Å^2^)

**Table:**

	*U*^11^	*U*^22^	*U*^33^	*U*^12^	*U*^13^	*U*^23^
O1	0.0133 (7)	0.0105 (7)	0.0283 (9)	0.0017 (6)	0.0008 (6)	−0.0010 (6)
N3	0.0091 (7)	0.0106 (8)	0.0179 (9)	0.0016 (6)	0.0007 (6)	0.0000 (7)
C3	0.0126 (9)	0.0118 (9)	0.0116 (9)	−0.0017 (8)	0.0006 (7)	−0.0008 (8)
C4	0.0085 (9)	0.0106 (9)	0.0147 (9)	0.0022 (7)	0.0011 (7)	−0.0001 (8)
N1	0.0082 (7)	0.0095 (8)	0.0124 (8)	0.0000 (6)	0.0008 (6)	−0.0003 (7)
C6	0.0107 (9)	0.0132 (10)	0.0095 (9)	0.0019 (8)	0.0004 (7)	0.0005 (8)
C2	0.0096 (9)	0.0132 (10)	0.0127 (9)	−0.0019 (7)	−0.0006 (7)	0.0005 (8)
C1	0.0094 (8)	0.0090 (9)	0.0109 (9)	0.0000 (7)	0.0020 (7)	0.0009 (7)
N2	0.0088 (8)	0.0113 (8)	0.0149 (8)	−0.0005 (6)	0.0005 (6)	0.0011 (7)
C5	0.0091 (9)	0.0114 (9)	0.0162 (10)	0.0010 (7)	0.0008 (7)	0.0018 (8)

### Geometric parameters (Å, °)

**Table:**

O1—C6	1.233 (3)	C4—H4B	0.9700
N3—C6	1.337 (3)	N1—C1	1.370 (3)
N3—H3A	0.8600	C6—C5	1.521 (3)
N3—H3B	0.8600	C2—N2	1.374 (3)
C3—C2	1.362 (3)	C2—H2A	0.9300
C3—N1	1.372 (3)	C1—N2	1.332 (2)
C3—H3C	0.9300	C1—C1^i^	1.465 (4)
C4—N1	1.472 (2)	C5—H5A	0.9700
C4—C5	1.523 (3)	C5—H5B	0.9700
C4—H4A	0.9700		
			
C6—N3—H3A	120.0	O1—C6—C5	120.75 (19)
C6—N3—H3B	120.0	N3—C6—C5	115.39 (18)
H3A—N3—H3B	120.0	C3—C2—N2	110.33 (17)
C2—C3—N1	106.55 (18)	C3—C2—H2A	124.8
C2—C3—H3C	126.7	N2—C2—H2A	124.8
N1—C3—H3C	126.7	N2—C1—N1	111.26 (17)
N1—C4—C5	111.30 (16)	N2—C1—C1^i^	124.5 (2)
N1—C4—H4A	109.4	N1—C1—C1^i^	124.2 (2)
C5—C4—H4A	109.4	C1—N2—C2	105.28 (17)
N1—C4—H4B	109.4	C6—C5—C4	111.26 (17)
C5—C4—H4B	109.4	C6—C5—H5A	109.4
H4A—C4—H4B	108.0	C4—C5—H5A	109.4
C1—N1—C3	106.58 (16)	C6—C5—H5B	109.4
C1—N1—C4	130.54 (16)	C4—C5—H5B	109.4
C3—N1—C4	122.78 (17)	H5A—C5—H5B	108.0
O1—C6—N3	123.84 (19)		

Symmetry codes: (i) −*x*+1/2, −*y*+3/2, −*z*+1.

### Hydrogen-bond geometry (Å, °)

**Table:**

*D*—H···*A*	*D*—H	H···*A*	*D*···*A*	*D*—H···*A*
N3—H3A···N2^ii^	0.86	2.22	3.055 (1)	164
N3—H3B···O1^iii^	0.86	2.13	2.967 (2)	165
C4—H4B···N2^i^	0.97	2.50	2.985 (2)	111
C5—H5B···O1^iii^	0.97	2.58	3.293 (3)	130

Symmetry codes: (ii) *x*+1/2, *y*−1/2, *z*; (iii) *x*, *y*+1, *z*; (i) −*x*+1/2, −*y*+3/2, −*z*+1.
