# 3,3′-(2,2′-Bi-1*H*-imidazole-1,1′-di­yl)dipropanamide. Corrigendum

**DOI:** 10.1107/S1600536810017526

**Published:** 2010-05-22

**Authors:** Y.-X. Zhi, J. Long, J.-Y. Chen, Y.-T. Ren, H.-Z. Liang

**Affiliations:** a Gemmological Institute, China University of Geosciences, Wuhan, Hubei 430074, People’s Republic of China; b China University of Geosciences, Engineering Research Center of Nano-Geomaterials of the Ministry of Education, Wuhan, Hubei 430074, People’s Republic of China; c Faculty of Materials Science and Chemical Engineering, Ningbo University, Ningbo 315211, People’s Republic of China

## Abstract

The list of authors in the paper by Zhi, Long, Chen & Ren [*Acta Cryst.* (2009), E**65**, o2008] is corrected and the acknowledgements are updated.

## Abstract 2

Corrigendum to *Acta Cryst.* (2009), E**65**, o2008.

In the paper by Zhi *et al.* (2009) ▶, the list of authors is incomplete. The correct full list of authors is given above. The acknowledgements are also updated and should read:
