# Tetra­kis-μ-l-alanine-κ^8^ *O*:*O*′-bis­[tetra­aqua­terbium(III)] hexa­perchlorate. Corrigendum

**DOI:** 10.1107/S1600536810027340

**Published:** 2010-07-17

**Authors:** Musa E. Mohamed, Deepak Chopra, K. N. Venugopala, Thavendran Govender, Hendrik G. Kruger, Glenn E. M. Maguire

**Affiliations:** a School of Chemistry, University of KwaZulu-Natal, Durban 4000, South Africa; b Department of Chemistry, Indian Institute of Science Education and Research, Bhopal 462 023, India; c School of Pharmacy and Pharmacology, University of KwaZulu-Natal, Durban 4000, South Africa

## Abstract

The surname of one of the authors and the affiliation of that author in the paper by Mohamed *et al.* [*Acta Cryst.* (2009), E**66**, m193–m194] are corrected.

## Abstract 2

Corrigendum to *Acta Cryst.* (2009), E**66**, m193–m194.

In the paper by Mohamed *et al.* (2009) ▶, the surname of one of the authors is incorrect, *viz.* Venugopal should appear as Venugopala, and the affiliation of the same author should be ‘School of Chemistry, University of KwaZulu-Natal, Durban 4000, South Africa’. The correct name and address are given above.
